# What Is the Role of the Gut Microbiota in Anastomotic Leakage After Colorectal Resection? A Scoping Review of Clinical and Experimental Studies

**DOI:** 10.3390/jcm13226634

**Published:** 2024-11-05

**Authors:** Georgios D. Lianos, Maximos Frountzas, Ilektra D. Kyrochristou, Panagiotis Sakarellos, Vasileios Tatsis, Gerasimia D. Kyrochristou, Christina D. Bali, Maria Gazouli, Michail Mitsis, Dimitrios Schizas

**Affiliations:** 1 Department of Surgery, University Hospital of Ioannina, 45110 Ioannina, Greece; glianos@uoi.gr (G.D.L.); electra.cyro@gmail.com (I.D.K.); tatsis.vasileios@hotmail.com (V.T.); ersie.cd@gmail.com (G.D.K.); cbali@uoi.gr (C.D.B.); mmitsis@uoi.gr (M.M.); 2 First Propaedeutic Department of Surgery, Hippocration General Hospital, School of Medicine, National and Kapodistrian University of Athens, 15772 Athens, Greece; 3 First Department of Surgery, Laikon General Hospital, School of Medicine, National and Kapodistrian University of Athens, 15772 Athens, Greece; panagiotissakarellos@gmail.com (P.S.); dschizas@med.uoa.gr (D.S.); 4 Laboratory of Biology, Department of Basic Medical Sciences, School of Medicine, National and Kapodistrian University of Athens, 15772 Athens, Greece; mgazouli@med.uoa.gr

**Keywords:** intestinal, microbiota, anastomotic leakage, colorectal

## Abstract

**Background:** Anastomotic leakage (AL) still remains a common complication after colorectal anastomosis that leads to increased morbidity and mortality. The gut microbiota has been hypothesized as one of the risk factors associated with anastomotic leakage. The aim of the present study was to summarize all existing clinical and experimental studies that evaluate the impact of intestinal microbiota on anastomotic leakage after colorectal resection. **Methods:** The present scoping review was designed according to PRISMA recommendations and a systematic search in Medline, Scopus, EMBASE, Clinicaltrials.gov, Google Scholar, and CENTRAL was conducted until September 2024. **Results:** Overall, 7 clinical and 5 experimental studies were included. A diminished α-diversity of the gut microbiota in patients suffering from AL was demonstrated. Specific microbe genera, such as *Lachnospiraceae*, *Bacteroidaceae*, *Bifidobacterium*, *Acinetobacter*, *Fusobacterium*, *Dielma*, *Elusimicronium*, *Prevotella*, and *Faecalibacterium*, seem to be associated with AL. However, specific genera, like *Prevotella*, *Streptococcus*, *Eubacterium*, *Enterobacteriaceae*, *Klebsiella*, *Actinobacteria*, *Gordonibacter*, *Phocaeicola*, and *Ruminococcus2*, seem to be protective against AL. Experimental studies highlighted that the Western diet seems to affect microbiota diversity and increases the AL rate, whereas anastomotic healing seems to be impaired by high metalloproteinase production and increased collagenase activity. **Conclusions:** The intestinal microbiota seems to play an important role in anastomotic leakage after colorectal resection. Specific interventions targeting the microbiota’s composition and the pathophysiological mechanisms by which it impairs anastomotic healing could diminish the risk for anastomotic leakage and improve clinical outcomes. However, future studies should be based on prospective design and eliminate heterogeneity.

## 1. Introduction

Nowadays, surgical resections of the colon or rectum due to malignant (colorectal cancer) or benign (inflammatory bowel disease or diverticulosis) conditions are very common procedures, which are usually followed by restoration of the continuity of the gastrointestinal tract with an anastomosis. Anastomotic leakage (AL) after colorectal resection is a common postoperative complication that appears in 2–20% of patients, depending on the location of the anastomosis. The incidence of AL in right anastomoses reaches up to 8%, whereas in low rectal anastomoses, the leakage rate can reach up to 20% [1,2]. In addition, anastomotic leakage is a serious complication that can affect the lives of patients, leading to a high mortality rate (up to 16% in rectal anastomotic leaks) and their oncologic outcomes, in the case of colorectal cancer (CRC), being associated with lower disease-free survival, overall survival, and cancer-specific survival [3]. Consensus for a widely-used definition of AL after colorectal resection was reached in 2020 and it was described as a defect of the integrity of the intestinal wall at the anastomosis or an abscess in the proximity of the anastomosis, indicating a communication between the intra- and extraluminal compartments [4,5].

Under these circumstances, a more precise definition for AL has been utilized in the literature and several more homogenous studies have investigated the potential modifiable and unmodifiable risk factors for AL after colorectal resection [6]. Male gender, increasing age, renal disease, obesity, American Society of Anesthesiology (ASA) classification > II, smoking, blood loss and intraoperative transfusion, malnutrition, and operative duration have been hypothesized as increasing the risk for AL after colon excision and anastomosis [7]. On the other hand, a small distance of the anastomosis from the anal verge, neo-adjuvant chemoradiotherapy, positive intraoperative leak test, and immunosuppressive therapy have been associated with high risk of AL in low rectal anastomoses [8]. Therefore, several preoperative strategies have been investigated for the optimization of the bowel conditions before resection and anastomosis, minimizing the risk for AL, such as the combination of mechanical bowel preparation (MBP) and oral antibiotics before surgery, which have been proven to reduce the risk of AL [9]. Nevertheless, the exact mechanism of action still remains unclear. The gut microbiota has been suggested as a potential factor that could affect the healing process in the microenvironment of the anastomosis, providing a potential therapeutic target for better clinical outcomes with a reduced AL rate. For example, a recent systematic review by Jørgensen et al. demonstrated the presence of one or more collagenase-producing bacteria, such as *Enterococcus* spp., *Pseudomonas* spp., *Klebsiella* spp., and *Proteus* spp. in the feces, mucosa, resected specimen, or drain fluid from patients with AL after colorectal resection [10].

However, there has not been a structured presentation of the existing clinical and experimental data about the potential effect of the gut microbiota on the AL rate after colorectal resection so far. The aim of this scoping review was to investigate the role of the gut microbiota in anastomotic failure after colorectal surgery, providing a thorough analysis of the existing literature based on clinical and experimental studies regarding the composition and possible mechanisms of interaction of the gut microbiota with factors affecting anastomotic healing. In this way, potential therapeutic targets for the improvement of postoperative outcomes can be suggested.

## 2. Material and Methods

### 2.1. Protocol and Registration

The present scoping review was designed according to the PRISMA extension for scoping reviews (PRISMA-ScR) recommendations [11]. This study was based on aggregated data that have been previously published in the international literature. Neither patient consent nor institutional review board approval were obtained as they are not required in this type of study. Our scoping review was registered in the Open Science Framework: http://www.osf.io/ (accessed on 22 October 2024) with the unique identifying number: osf.io/ap8mk.

### 2.2. Study Types

The eligibility criteria were predefined by the authors and no data restriction was applied during searching. All articles written in the Latin alphabet were included in the present study, as well as articles written in other languages, if they could be translated using the Google Translate service. Observational clinical trials and experimental studies that directly investigated the role of the gut microbiota in the AL rate after colorectal anastomosis and presented specific microbiome species and their mechanism of action in patients or experimental animal models were included in our study. Several studies in the literature that assumed the impact of the gut microbiome on colorectal anastomoses as a secondary hypothesized outcome due to its interaction with other agents, such as perioperative antibiotic or prebiotic administration, were excluded from the present study.

### 2.3. Information Sources and Search

Medline (1966–2021), Scopus (2004–2021), EMBASE (1980–2021), Clinicaltrials.gov (2008–2021), Google Scholar (2004–2021), and Cochrane Central Register of Controlled Trials CENTRAL (1999–2021) were scanned by two authors until 30 September 2024. In addition, the chance of including all available articles that met the inclusion criteria was maximized by searching the references of articles that were retrieved in full text. Any disagreements between reviewers were resolved by a third reviewer. The main search strategy included the following text words: (“gut” and/or “microbiome” or “microbiota” and “colorectal” and “anastomotic” and “leak” or “leakage”).

**Figure 1:**
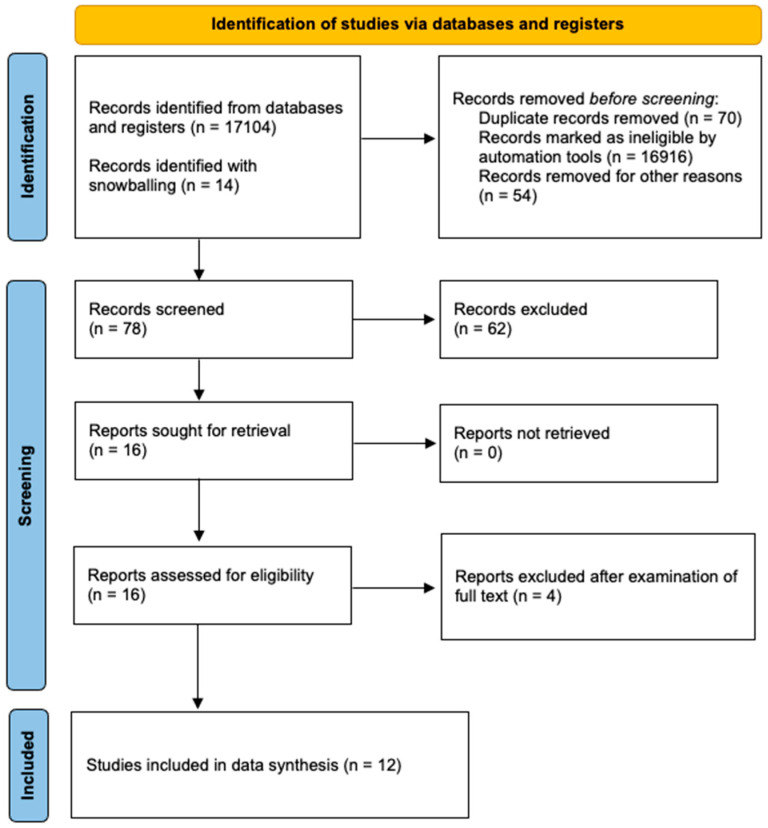
PRISMA flowchart of study selection process.

Study selection was performed in three consecutive stages: First, duplicate publications were removed and then the titles and abstracts of all electronic articles that were retrieved in the search were evaluated in terms of eligibility. Second, the full texts of all articles that met the inclusion criteria were downloaded and all observational clinical trials along with experimental studies were selected. The study search and data tabulation were conducted by two authors on similar pre-defined forms. A consensus of all authors resolved any possible conflicts after retrieving all available data. The article selection process is depicted in Figure 1.

### 2.4. Data Synthesis

The great heterogeneity among the data of the clinical and experimental studies necessitated their structured presentation in four tables. Table 1 describes the characteristics of the included clinical trials, such as their type, the number of patients enrolled, and their inclusion, as well as their exclusion criteria. Table 2 depicts the demographic characteristics of the patients enrolled in the clinical studies, like their age, their gender, their body mass index (BMI), the administration of neo-adjuvant chemoradiotherapy, and the number of patients with AL after colorectal resection. Table 3 summarizes the methods and outcomes of the included clinical trials, such as the type of microbiome sample gathered, the method of maintenance, the methods of DNA extraction and sequencing, as well as the study findings. On the other hand, Table 4 presents the study design of the experimental studies included, the number of experimental animals enrolled, the methods of DNA extraction and sequencing, as well as the study findings.

## 3. Results

### 3.1. Included Studies

Overall, 12 studies were included in the data synthesis of the present scoping review [12,13,14,15,16,17,18,19,20,21,22,23]. Seven studies were prospective clinical trials that investigated the impact of the gut microbiota on the AL rate of patients undergoing colorectal resection [12,13,14,15,16,17,18]. On the other hand, five studies demonstrated experimental models of colorectal anastomoses that investigated the effect of the gut microbiota on AL and the molecular interactions between specific microbes and anastomotic healing mechanisms [19,20,21,22,23]. Four studies were excluded after full text retrieval because two of them investigated the impact of perioperative antibiotics and prebiotics on the AL rate, hypothesizing the role of the microbiome in this relationship as such agents could alter the gut microbiota, but the causative relationship between the microbiome and anastomotic leakage was not proven by specific investigations about different microbes and their way of action [24,25]. Another excluded study presented mixed outcomes about the effect of the gut microbiota on several postoperative outcomes after colorectal resection without providing specific data about anastomotic leakage [26], and the other excluded study demonstrated the role of *Clostridium difficile* infection on pouch failure in patients undergoing total proctocolectomy due to ulcerative colitis [27].

### 3.2. Observational Clinical Trials

In 2016, van Praagh et al. designed a prospective pilot study to compare the intestinal microbiota of CRC patients with AL after colorectal resection compared to patients without AL. They demonstrated diminished α-diversity (the species of microbes constituting the gut microbiota) in patients with AL compared to patients without AL (*p* = 0.037). In addition, higher abundance of the family *Lachnospiraceae* was observed in the AL group compared to the non-AL group and the presence of this family was associated with higher BMI of patients [17].

However, in 2019, van Praagh et al. published their main prospective observational study that investigated the association of the gut microbiota with AL after colorectal resection with the utilization of a healing agent, called c-seal, or not. In colorectal anastomoses with c-seal, diminished α-diversity in the gut microbiota was observed, but without differences between the AL and non-AL groups, apart from higher abundance of the family *Blautia* in the AL group. On the contrary, in colorectal anastomoses without c-seal, there were higher abundances of *Lachnospiraceae* and *Bacteroidaceae* in the AL group compared to increased abundances of the families *Prevotella*, *Streptococcus*, and *Eubacterium* in the non-AL group [18].

In 2020, Mima et al. tried to prove the specific CRC microbes that lead to an increased AL rate and performed shotgun metagenomics using quantitative PCR looking for *Fusobacterium nucleatum*, pks (+) *E. coli*, *Enterococcus faecalis*, and the genus *Bifidobacterium* in tumor and healthy tissue samples collected intraoperatively 5–10 cm from the tumor edge. AL was associated with a higher abundance of the genus *Bifidobacterium* (OR 3.96, 95% CI 1.5–10.51), which was also characterized as an independent risk factor of AL [14]. Moreover, Palmisano et al. aimed to define a peculiar microbiota composition collected preoperatively in fecal samples that could be associated with higher incidence of AL. Under these circumstances, they demonstrated higher abundances of *Acinetobacter lwoffii*, *Acinetobacter johnsonii*, *Hafnia alvei*, and *Odoribacter ianeus* in the AL group, whereas lower abundances of *Barnesiella intestinihominis* and *Faecalibacterium prausnitzii* were observed in AL patients [15].

Shi et al. extracted DNA from tumor tissue samples intraoperatively collected and proved that α-diversity was different between the AL and non-AL groups [16]. In detail, patients demonstrating AL after surgery demonstrated poorer α-diversity, with predominance of *Lachnospiraceae*, *Bacteroidaceae*, and *Fusobacterium*. On the contrary, patients with an uncomplicated postoperative course showed higher abundances of *Enterobacteriaceae*, *Klebsiella*, unclassified-k-no rank-d-bacteria, *Burkholderiaceae*, and *Ralstonia*. Furthermore, based on previous data regarding the role of *Fusobacterium nucleatum* (Fn) in cancer and inflammation-related dysbiosis [28,29], the researchers conducted a PCR search for Fn genetic material and showed that 87.5% of the AL patients were positive for Fn, which was also an independent risk factor of AL (OR 22.308, AUC 71.94%, *p* = 0.048).

Concerning β-diversity, Hernandez-Gonzalez et al. demonstrated that the non-AL group had higher abundances of *Actinobacteria* and *Gordonibacter*, whereas AL patients showed higher abundances of *Dielma* and *Elusimicronium* [12]. They also focused on the differences in microbes isolated from different types of samples, such as feces, mucus, or peritoneal fluid, concluding that the α-diversity of the drain fluid differed from that of fecal/mucosal samples and was higher in feces than in the other types of samples.

Apart from the type of sample, there are also other important parameters for the investigation of the specific role of the gut microbiota in AL after colorectal resection. In a recent prospective trial, Lehr et al. aimed to highlight the differences between certain time points of microbiome sample collection regarding the time of surgery and the differences between certain bowel locations for tissue sampling regarding the location of the tumor [13]. They demonstrated variability in the microbiota depending on the time of tissue sampling, with the greatest variability before surgery, but not depending on the location of tissue sampling. In addition, the α-diversity of the microbiota decreased over time, but no difference was observed between the AL and non-AL groups. Finally, *Prevotella* and *Faecalibacterium* presented higher abundances in the AL group, whereas *Phocaeicola* and *Ruminococcus2* presented higher abundances in the non-AL group. The predictive value of these genera for AL revealed an AUC of 0.802 (*p* = 0.0013).

### 3.3. Experimental Studies

Christley et al. described a rat anastomotic model to investigate the genetic differences between preoperative and postoperative isolates of *E. faecalis* in the intestinal wall. They reported higher postoperative collagenolytic activity of *E. faecalis* at anastomotic sites, even though preoperative and postoperative *E. faecalis* isolates were nearly identical in the fsr-gelE-sprE region, which is responsible for the regulation and production of collagenase activity [21]. This observation implied that not only genetic but also still unknown epigenetic factors might have a great impact on microbiome formation, which could therefore be altered to achieve better clinical outcomes.

In 2023, Hajjar et al. created a murine model to assess the influence of the gut microbiota in anastomotic healing and gut barrier integrity. After transplanting the feces of patients demonstrating or not AL after colorectal surgery, they tried to identify the collagenolytic strains of *Enterococcus faecalis* that were predominant at the anastomotic sites and the mechanisms through which they may lead to AL [22]. They concluded that higher abundances of *Alistipes onderdonkii* and *Parabacteroides goldsteinii* increased AL incidence, although enemas with *P. goldsteinii* improved healing in the in vitro model by exerting an anti-inflammatory effect. An upregulation of mucosal macrophage inflammatory protein-1α (MIP-1α), MIP-2, monocyte chemoattractant protein-1 (MCP-1), and interleukin-17A/F (IL-17A/F) was observed preoperatively in mice receiving FMT (fecal microbiota transplantation) from AL patients. All of these metalloproteinases are key contributors to the degradation of the intestinal mucosa, leading to AL, as well as promoting metastasis in CRC patients.

Another mechanism that has been correlated with AL is the exposure of the intestinal mucosa to oxygenated air during surgery. Zamorano et al. created a rat model to assess the effect of oxygen exposure on mucous-degrading bacteria, which increase AL incidence [19]. Interestingly, they found that oxygen exposure led to significantly poorer α-diversity after surgery, but no changes were observed in the relative abundances of obligate anaerobic bacteria, apart from facultative anaerobes. Therefore, *Gemella palaticanis* and *Lactobacillus* spp. were the species that best represented the AL phenotype.

The Western diet (WD) has been also investigated as a risk factor for AL. In 2020, Hyoju et al. demonstrated that WD was associated with larger stool volumes in the first 5 postoperative days (POD) and altered bowel function, which interfered with anastomotic healing [23]. When the test animals were not shifted from a high-fat diet to a low-fat one perioperatively, a significant decrease in the abundance of *Bacteroidetes* in the luminal content was observed, along with an increase in the expelled stool and the emergence of *Proteobacteria* in mucosal samples. Moreover, WD mice demonstrated a higher abundance of collagenolytic populations of *Enterococcus* remaining even 1 month postoperatively. A shift from WD to standard diet (SD; low fat/high fiber) 2 days before surgery improved overall diversity and decreased postoperative collagenolytic populations of *Enterococcus*.

Boatman et al. also reported that shifting mice preoperatively from WD to SD achieved a lower rate of AL [20]. In addition, α-diversity, which was proven to be an independent factor for AL, was significantly higher in the SD compared to WD mice, with mean Chao1 indexes of 172.6 ± 4.8 for SD and 150.6 ± 4.7 for WD (F = 10.7, *p* = 0.0022). *Bacteroides*, *Akkermansia*, *Lachnospiraceae* spp., and other species with high collagenolytic action were significantly associated with the WD axis position (*p* < 0.04). Finally, a positive correlation was reported between *Bacteroides* and overall fat percentage, saturated fat, mono- and polyunsaturated fats, as well as simple sugar content.

## 4. Discussion

Anastomotic leakage after colorectal resection still remains a common and serious complication requiring predictive measures and potential interventions to improve clinical outcomes. The gut microbiota seems to play an important role in the pathophysiology of AL after colorectal anastomosis, as summarized in the present scoping review. Existing observational clinical trials in the field indicate a diminished α-diversity of the gut microbiota in patients suffering from AL. In addition, specific microbe genera, such as *Lachnospiraceae*, *Bacteroidaceae*, *Bifidobacterium*, *Acinetobacter*, *Fusobacterium*, *Dielma*, *Elusimicronium*, *Prevotella*, and *Faecalibacterium*, seem to be associated with AL. On the contrary, specific genera, like *Prevotella*, *Streptococcus*, *Eubacterium*, *Enterobacteriaceae*, *Klebsiella*, *Actinobacteria*, *Gordonibacter*, *Phocaeicola*, and *Ruminococcus2*, seem to be protective against AL. Interestingly, the type and timing of tissue sampling seem to play a crucial role in the current research field, as the α-diversity of the gut microbiota is higher in feces than in drain/mucosal samples and at the time point before surgery. On the other hand, experimental studies highlight the different pathogenetic mechanisms through which the gut microbiota affects anastomotic healing. The Western diet seems to affect microbiota diversity and increase the AL rate. In addition, anastomotic healing seems to be impaired by high metalloproteinase production and increased collagenase activity, which is regulated not only by genetic factors of the gut microbiota but also epigenetic ones.

Several pathogenetic mechanisms have been proposed about how the intestinal microbiota could lead to anastomotic leakage after colorectal anastomosis, including dysbiosis, bacterial translocation, biofilm formation, immune system modulation, and disturbance of tissue metabolism. First of all, imbalance in the intestinal microbial community, which is called dysbiosis, with increases in the abundances of harmful genera and decreases in the abundances of beneficial microbes, as discussed before, could lead to impaired healing at the anastomotic site due to exert collagenase secretion, which degrades the collagen matrix of the anastomosis [30]. Moreover, dysbiosis seems to damage the intestinal mucosal barrier, which leads to bacterial translocation at the anastomotic site. It creates a local inflammatory reaction that is mediated by pro-inflammatory biomolecules, such as tumor necrosis factor-a (TNF-a) and interleukin-6 (IL-6), which could delay wound healing, disrupt angiogenesis, and increase tissue necrosis [31]. Biofilm formation is another mechanism through which certain genera –*Enterococcus* and *Staphylococcus*–could increase the risk for AL at the anastomotic site, as it creates a physical barrier that interferes with collagen deposition and re-epithelization, both essential for proper tissue healing, while it enhances pathogen resistance to immune clearance and antibiotics [32]. Furthermore, harmful intestinal bacteria seem to modulate the local immune system by activating macrophages and neutrophils, which release proteolytic enzymes and reactive oxygen species that degrade healing tissue at the anastomotic site [33]. Finally, dysbiosis has been associated with decreased formation of short-chain fatty acids, which have anti-inflammatory and epithelial barrier-strengthening effects, promoting anastomotic healing, whereas nutrient absorption is impaired, leading to delayed collagen synthesis [34].

### 4.1. Factors Affecting the Gut Microbiota

An example of such epigenetic alterations could be the impact of morphine on *E. faecalis* at the anastomotic site. Shakhsheer et al. investigated the growth, collagenase production, and adherence ability of *E. faecalis* in rats demonstrating AL after being administered morphine as analgesia following colorectal surgery and primary anastomosis [35]. Morphine seemed to increase collagenase production concerning cell density in four distinct strains of *E. faecalis* tested, indicating that postoperative opioids might play a role not only in postoperative ileus but also in AL after colectomy. Another epigenetic alteration that could affect the impact of the microbiota on colorectal anastomotic healing could be intraoperative tissue oxygen exposure, which results in the loss of some protective obligate anaerobes, such as *Bacteroides*, and increases in the abundances of degrading facultative anaerobes, such as *Enterococcus*, in rat models investigating the mucosa-associated microbiome (MAM) [36]. Nevertheless, contradictory outcomes were observed in other animal intervention studies that noted increased remodeling activity by higher levels of metalloproteinases (MMP) 1 and MMP 9 in segmentally de-vascularized groups treated with hyperbaric oxygen therapy (HBOT). HBOT therapy was also associated with fewer septic complications, prevention of adhesions, increased angiogenesis, and higher concentrations of hydroxyproline, advocating greater collagen deposition and better healing of the anastomosis [37,38].

Another extrinsic factor that could alter the gut microbiota and further affect AL after colorectal anastomosis is mechanical bowel preparation, which is now the standard of care according to the Enhanced Recovery After Surgery (ERAS) protocol in colorectal surgery, especially in left-sided and rectal resections [39]. Multiple literature reports have proven that preoperative mechanical bowel preparation alone demonstrates no benefit in reducing AL after elective colorectal surgery. A randomized controlled trial with 1431 patients conducted by Contant et al. showed that mechanical bowel preparation alone did not result in decreased AL incidence [40]. This finding was also confirmed by a systematic review and meta-analysis of RCTs including 4859 patients [41]. Nevertheless, Scarborough et al. indicated that mechanical bowel preparation enhanced the delivery of oral antibiotics at the bowel mucosa and, therefore, these two practices cannot be assessed separately. Indeed, among almost 5000 patients, combined mechanical bowel preparation and oral antibiotic administration diminished the percentage of AL from 5.7% to 2.8% compared to patients receiving no preparation [42]. In addition, a double-blind, placebo-controlled randomized trial by Kotzampassi et al. demonstrated the protective effect of probiotics (*Lactobacillus acidophilus*, *L. plantarum*, *Bifidobacterium lactis*, and *Saccharomyces boulardi*) in anastomotic leakage (8.8% in the placebo group vs. 1.2% in probiotics group, *p* = 0.031) after colorectal resection [43].

Apart from extrinsic factors that could interfere with the impact of the gut microbiota on AL after colorectal resections, the type of tissue sample could also play an important role, as indicated by the present study as well. As one could easily understand, the fecal microbiota, also called the luminal microbiota (LM), is prone to mechanical bowel preparation and oral antibiotic treatment. On the contrary, mucosal intestinal samples gathered during surgery could provide a much more accurate estimation of the microbiota independent of all the aforementioned factors. This type of microbiome is called the mucosa-associated microbiome (MAM) and constitutes a microbial community of bacteria, viruses, fungi, and archaea under local and host-related differences along the length of the colon [44]. Evaluating the MAM might be more accurate than the LM, although it requires a colonoscopy before surgery to obtain the sample or intraoperative selection of tissue, both more difficult than the simple collection of fecal samples before or after surgery.

### 4.2. Strengths and Weaknesses

To the best of our knowledge, the present scoping review represents the first structured summary of all of the available clinical and experimental studies that investigate the impact of the gut microbiota on the postoperative AL rate after colorectal resection and provide direct data about specific microbiome populations and potential pathophysiologic mechanisms that affect the anastomotic healing process. In addition, wide inclusion criteria were applied during the search procedure, hence maximizing the chance of including all available studies related to our topic. The search and selection of the included studies were performed by two independent researchers, limiting selection bias. Another advantage is the predefined methodological protocol that was followed during the interpretation of the present meta-analysis, which is also registered in an international database (Open Science Framework), giving every reviewer the chance to assess its methodological quality.

Nevertheless, the present study has certain limitations. Firstly, the great heterogeneity among studies regarding the type of tissue sample for microbiome analysis and the timing of tissue sampling, along with the different methods for DNA extraction and synthesis, did not allow a quantitative synthesis of the results, even with subgroup analysis among clinical and experimental studies. Moreover, the small number of included patients or test animals for clinical and experimental studies, respectively, as well as their observational design, resulted in low methodological quality. Therefore, a risk of bias assessment of the eligible studies was not considered obligatory. However, future experimental and clinical studies could eliminate these drawbacks to enhance the quality of evidence in the current research field.

### 4.3. Clinical Practice and Future Research

In the clinical setting, specific patient characteristics should always be considered. Right colonic anastomoses present a lower risk for leakage compared to left-sided colorectal or coloanal anastomoses (1–8% vs. 5–10%), implicating the use of intraoperative protective measures after left-sided anastomoses, such as defunctioning loop-ileostomy, which seems to be protective against anastomotic leakage [45,46]. Nevertheless, the present scoping review highlights that the gut microbiota could play an important role in the prediction of AL in patients undergoing colorectal resection. Under these circumstances, this could lead to further therapeutic interventions to decrease the AL rate. First of all, better targeting of collagenase-inducing pathogens identified by microbiome analysis, such as *Pseudomonas aeruginosa* and *Enterococcus faecalis*, could potentially improve clinical outcomes, such as AL and surgical site infections (SSIs) [47]. In addition, preoperative dietary prehabilitation with a high-fiber diet could reverse the dysbiotic effect of the Western diet on the intestinal microbiota, diminishing the risk for AL [48]. This could be further enhanced by the utilization of probiotics, such as *Lactobacillus plantarum*, *Lactobacillus rhamnosus*, *Lactobacillus casei*, *Lactobacillus reuteri*, *Bifidobacterium longum*, and *Bifidobacterium breve*, which seem to produce anti-inflammatory cytokines (IL-4, IL-10, IL-11, and IL-13), while de-activating pro-inflammatory cytokines (IL-1, IL-6, and TNF-α), reducing inflammatory reactions throughout the gastrointestinal tract [49]. Furthermore, Jin et al. performed FMT, using feces of patients with CRC who demonstrated AL or not after surgery, in a group of rats [50]. They reported that the gut microbiota was significantly different between AL and non-AL patients, but also the test animals transplanted with the fecal microbiome from non-AL patients demonstrated increased collagen synthesis and upregulation of the tumor growth factor (TGF)-β/Smad signaling pathways in vitro and in vivo. Nonetheless, the gut microbiota is prone to several extrinsic factors that are extremely difficult to compare among study groups of patients, such as dietary habits, medicinal agents (metformin, levothyroxine, PPIs), stress, and exercise [51,52]. Therefore, diminishing heterogeneity should be a primary goal of future clinical studies conducted in different geographical regions of the world where people adopt different ways of life that directly interact with their gut microbiome. Last but not least, having an adequate microbiome helps, but it might not be sufficient on its own to protect the individual on whom a low left or subperitoneal anastomosis needs to be performed. So, especially in people who have undergone neoadjuvant treatment with radiotherapy, a temporary ileostomy is better, even if a second recanalization operation will be necessary.

## 5. Conclusions

The gut microbiota as a predictive factor of postoperative outcomes is being increasingly established in colorectal surgery. The need for more DNA sequencing research on humans to validate the existing experimental data is apparent. In addition, the determination of the exact mechanisms through which the different genera contribute to the multifactorial process of AL and other inflammatory complications could be very important. Taking advantage of the existing evidence, well-designed clinical studies could assess the specific applications of gut microbiota research on human outcomes and determine potential therapeutic interventions.

## Figures and Tables

**Table 1:** Characteristics of clinical studies investigating the role of the gut microbiome in anastomotic leakage after colorectal surgery.

Year; Author	Type of Study	No of Patients	Inclusion Criteria	Exclusion Criteria
2016; van Praagh	Prospective pilot study	16	-Elective colorectal surgery ->18 years old -ASA < 4 -Mechanical bowel preparation -No clinical signs of peritonitis	-Major surgical/interventional procedure 30 days prior to surgery -Psychological/familial/sociological/geographical conditions hampering compliance
2019; van Praagh	Prospective	60	-Elective colorectal surgery ->18 years old -ASA < 4 -No clinical signs of peritonitis -Mechanical bowel preparation + oral bowel preparation -Preoperative antibiotic prophylaxis	-Major surgical/interventional procedure 30 days prior to surgery -Psychological/familial/sociological/geographical conditions hampering compliance
2020; Mima	Prospective	256	-Colorectal cancer -Mechanical bowel preparation -Preoperative antibiotic prophylaxis	N/A
2020; Palmisano	Prospective	21	->18 years old -Colorectal cancer	-Antibiotics 1 month before surgery -Previous gastrointestinal surgery -Diverting stoma -Inflammatory bowel disease -Hartmann procedure and abdominoperineal resection
2022; Shi	Prospective	45	-Colorectal cancer -Colorectal resection -First Affiliated Hospital of Harbin Medical University	-<18 years -Emergent surgery -Protective stoma -Underlying severe disease -Neo-adjuvant chemotherapy
2023; Hernandez-Gonzalez	Prospective	111	-Mechanical bowel preparation -Elective surgery -Preoperative antibiotic prophylaxis	N/A
2024; Lehr	Prospective	16	-Rectal cancer -Low anterior resection + anastomosis -Oral preoperative and intraoperative antibiotics	N/A

ASA, American Society of Anesthesiologists; N/A, not available.

**Table 2:** Demographic characteristics of patients enrolled in clinical studies investigating the role of the gut microbiome in anastomotic leakage after colorectal surgery.

Year; Author	Anastomotic Leakage	Age (Years)	Gender	Body Mass Index (kg/m^2^)	Neo-Adjuvant Therapy
2016; van Praagh	8 vs. 8	66.5 vs. 66.5	7 males + 1 female vs. 7 males + 1 females	25.4 vs. 30.1	3 vs. 3
2019; van Praagh	11 vs. 49	63.4 ± 10.4 vs. 63.4 ± 10.4	8 males + 2 female vs. 37 males + 12 females	26.5 vs. 29.5	7 vs. 36
2020; Mima	29 vs. 227	≥75 years: 10 vs. 72	25 males + 4 females vs. 127 males + 100 females	>30: 29 (100%) vs. 9 (4%)	N/A
2020; Palmisano	5 vs. 16	76 vs. 74.5	3 males + 2 females vs. 8 males + 8 females	24.76 vs. 24.16	2 vs. 4
2022; Shi	16 vs. 29	N/A	N/A	N/A	N/A
2023; Hernandez-Gonzalez	10 vs. 101	70.6 ± 11.6 vs. 73.2 ± 10.8	7 males + 3 females vs. 54 males + 47 females	25.5 vs. 24.8	N/A
2024; Lehr	5 vs. 11	60 ± 8	12 males + 4 females	N/A	8

N/A, not available.

**Table 3:** Methods and outcomes of clinical studies investigating the role of the gut microbiome in anastomotic leakage after colorectal surgery.

Year; Author	Sample	Maintenance	DNA Extraction	DNA Sequencing	Outcomes
2016; van Praagh	Anastomotic donuts	N/A	Bead-beating method via the Precellys 24 tool	16s rRNA sequencing via MiSeq platform (2 × 300)	-↓ α-diversity in AL group (*p* = 0.037) -↑ Family Lachnospiraceae in the AL group -Ruminococcus → mucin-degrading qualities -Family Lachnospiraceae → ↑ BMI
2019; van Praagh	Proximal anastomotic donuts	N/A	Bead-beating method via the Precellys 24 tool	16s rRNA sequencing via MiSeq platform (2 × 300)	-Non-c-seal group: ↑ Lachnospiraceae and Bacteroidaceae in AL group vs. ↑ Prevotella, Streptococcus, and Eubacterium in non-AL group -C-seal group: ↓ α-diversity without differences between AL and non-AL groups, ↑ Blautia abundance in AL group
2020; Mima	-Tumor tissue -Intraoperative 5–10 cm from tumor edge	Immediately frozen at −80 °C via liquid nitrogen	QIAamp DNA Mini Kit (Qiagen)	Shotgun metagenomics with PCR for *Fusobacterium nucleatum*, pks (+) *E. coli*, *Enterococcus faecalis*, and genus Bifidobacterium	-↑ Bifidobacterium → ↑ AL (OR 3.96, 95% CI 1.5–10.51) -No significant relationship between AL and the rest of the microbes -Bifidobacterium → independent risk factor of AL
2020; Palmisano	Stool	N/A	NucliSENS easyMAG semiautomatic system and Nanodrop 100	Sequential real time PCR	-↑ Acinetobacter lwoffii, Acinetobacter johnsonii, Hafnia alvei, and Odoribacter ianeus in AL group -↓ Barnesiella intestinihominis and Faecalibacterium prausnitzii in AL patients
2022; Shi	Intraoperative tumor tissue	Immediately frozen in liquid nitrogen	TRIzol Reagent	-16s rRNA sequencing via Illumina LiSeq platform (2 × 300) -Shotgun with PCR targeting *Fusobacterium nucleatum*	-α-diversity different between the AL and non-AL group -β-diversity → ↑ Lachnospiraceae, Bacteroidaceae, and Fusobacterium in the AL group and ↑ Enterobacteriaceae, Klebsiella, unclassified-k-norank-d-bacteria, Burkholderiaceae, and Ralstonia in the non-AL group -AL → ↑ Fn (87.5% vs. 48.28%) -Fn → independent risk factor of AL (OR 22.308, AUC 71.94%, *p* = 0.048)
2023; Hernandez-Gonzalez	-Fecal 2–3 days prior surgery -Intraoperative tissue of healthy margins proximal and distal to the tumor	Immediately frozen at −80 °C	Speedtools tissue	16s rRNA sequencing via MiSeq platform (2 × 300)	-α-diversity → ↑ in feces than in biopsy samples -Νon-AL group → ↑ Actinobacteria and Gordonibacter -AL group → ↑ Dielma and Elusimicronium -α-diversity of drain fluid different from fecal/mucosal samples
2024; Lehr	-Biopsy before, during and after surgery -Before tumor, at the tumor site, and after junction	Frozen in liquid nitrogen and stored at −80 °C	FastPrep-24 Instrument	16s rRNA sequencing via MiSeq platform (2 × 300)	-Variability in the microbiota depending on time (greatest before surgery) but not location -Microbiota α-diversity decreased over time, but no difference between AL and non-AL groups - Prevotella → ↑ in AL group, Phocaeicola → ↑ in non-AL group and Enterococcus most abundant after surgery -↑ Prevotella and Faecalibacterium in AL group and ↑ Phocaeicola and Ruminococcus2 in non-AL group -AUC of 0.802 (*p* = 0.0013) for prediction of AL

N/A, not available; PCR, polymerase chain reaction; AL, anastomotic leakage; Fn, Fusobacterium nucleatum; AUC, area under the curve.

**Table 4:** Experimental studies investigating the role of the gut microbiome in anastomotic leakage after colorectal surgery.

Year; Author	Study Design	No of Animals	Sample	Maintenance	DNA Extraction	DNA Sequencing	Outcomes
2020; Christley	Rat model (isolates of *Enterococcus faecalis*)	3	Tissue swabs preoperatively and on POD6	Frozen stocks on tryptic soy broth plates and incubated	OpGen HMW DNA Isolation Kit	ARGUS1 Whole Genome Mapping System	-↑ collagenolytic activity of *E. faecalis* on anastomotic sites -Difference between preoperative and postoperative genome sequences of *E. faecalis*, but nearly identical in the fsr-gelE-sprE region that is responsible for regulation of collagenase activity -No obvious genetic differences in the fsr-gelE-sprE region that regulates GelE expression
2020; Hyoju	Murine model (high-fat Western-type diet for 6 weeks)	124	-Stool samples -Colonic luminal content -Tissue samples on POD 1, 3, 7, 13, 16, 24 and 28	N/A	DNeasy PowerSoil HTP 96 Kit	16s rRNA sequencing	-WD → ↑ stool volumes in the first 5 POD -WD altered intestinal microbiota prior and after surgery: ↓ Bacteroidetes and ↑ Proteobacteria -WD → ↑ Enterococcus remaining for 1 month postoperatively, while cleared from SD mice’s intestinal lumen on POD -β-diversity not normal in WD mice, while in SD mice, normal within 2 weeks -Collagenolytic populations of Enterococcus remain in WD group even after POD5. -WD to SD shift → restored Bacteroidetes population in WD mice, improved overall diversity, and decreased postoperative collagenolytic Enterococcus population
2023; Boatman	Murine model (WD or SD before colorectal surgery and anastomosis)	64	Fecal samples	Frozen at −80 °C	DNeasy PowerSoil Pro kit	16s rRNA sequencing	-WD to SD shift → ↓ AL -α-diversity → ↑ SD vs. WD mice at baseline (*p* = 0.0022) -Difference in β-diversity between 2 groups -↓ α-diversity → independent risk factor of AL -Bacteroides → ↑ total fat, saturated fat, mono- and polyunsaturated fat, and simple sugar content and ↓ total carbohydrates and polysaccharides -FMT → AL alteration
2023; Hajjar	Murine AL model (FMT from CRC patients with AL or not)	3	Feces and tissue swabs preop and on POD6	Frozen stocks on tryptic soy broth plates and incubated overnight at 37 °C	Illumina MiSeq platform	-Genomes of seven *E. faecalis* strains using Bowtie2 version -Whole Genome Map with restriction enzyme NcoI for *E. faecalis*	-↑ *Alistipes onderdonkii* and *Parabacteroides goldsteinii* → ↑ AL -*P. goldsteinii* → ↑ healing -FMT from AL patients → ↑ mucosal MIP-1α, MIP-2, MCP-1, and IL-17A/F
2023; Zamorano	Rat model (comparing α- and β-diversity of between AL and non-AL groups)	12	Mucosal tissue after necropsy on POD6	Frozen at −80 °C	EZNA DNA/RNA Isolation kit	16s rRNA sequencing	-↑ dominance and ↓ heterogeneity of microbiota in AL -No change in anaerobic bacteria, but ↑ facultative anaerobes -*Gemella palaticanis* and *Lactobacillus* spp. → best represented the AL phenotype -Non-AL →↑ Helicobacter rodentium, *Lactobacillus murinus*, *Ligilactobacillus* spp., *Lactobacillus reuteri*, *Rhodospirillales* spp., UCG_005 spp., *Clostridium sensu stricto* spp., *Muribaculaceae* spp., and *Muribaculaceae* spp.

N/A, not available; PCR, polymerase chain reaction; AL, anastomotic leakage; WD, Western-type diet; SD, standard-type diet; FMT, fecal microbiota transplantation; CRC, colorectal cancer; POD, postoperative day; MIP, macrophage inflammatory protein; MCP, monocyte chemoattractant protein; IL, interleukin.
